# Optimized Yamanaka factors combined with TERT gene therapy for enhanced anti-aging effects

**DOI:** 10.1016/j.gendis.2025.101669

**Published:** 2025-05-12

**Authors:** Mengmeng Jiang, Qianqian Xu, Zhengzhi Wu

**Affiliations:** a Shenzhen Institute of Translational Medicine, The First Affiliated Hospital of Shenzhen University, Shenzhen Second People's Hospital, Shenzhen, Guangdong 518035, China; b Department of Neurology, The First Affiliated Hospital of Shenzhen University, Shenzhen Second People's Hospital, Shenzhen, Guangdong 518035, China; c Wu Zhengzhi Academician Workstation, Ningbo College of Health Sciences, Ningbo, Zhejiang 315800, China

Aging is a natural process characterized by the progressive decline in tissue and cellular functions, while recent studies indicated the potential reversibility of aging. Gene reprogramming is a typical method that involves cellular reprogramming. The Yamanaka factors, comprising Oct4, Sox2, Klf4, and c-Myc (termed OSKM), have been shown to convert senescent cells into induced pluripotent stem cells and mitigate signs of premature aging in presenile mice. Despite these promising results, the continuous expression of Yamanaka factors induces teratomas and severe mortality, primarily due to the oncogenic properties of c-Myc. It is well-established that c-Myc functions as a key transcription factor for activating the telomerase gene (TERT), and the absence of c-Myc may impede telomere elongation, thereby compromising anti-aging effects. To address these issues, this study employed an optimized Yamanaka factor (Oct4-Sox2-Klf4, OSK) that excludes c-Myc in conjunction with TERT gene therapy. Through a series of experimental validations, we confirmed that the co-expression of the OSK and TERT genes significantly enhanced the expression of genes associated with youth while concurrently reducing the levels of senescence-associated genes. Consequently, this combined gene therapy represents a promising therapeutic strategy for extending lifespan and addressing aging-related diseases.

Aging is influenced by a variety of interconnected molecular and cellular mechanisms, including genomic instability, cellular senescence, telomere attrition, and stem cell exhaustion.^1^ The emerging evidence indicates that targeting the aging process may hold promise for the development of therapeutic interventions for age-related diseases. Cell reprogramming technology, notably the identification of the Yamanaka factors, challenges the long-held belief that the entirely differentiated somatic cells are irreversibly turned into pluripotent cells, thus providing an innovative approach for generating stem cells. There is a periodical transgene of OSKM into premature aging mice that markedly extends their lifespan, suggesting a possibility of counteracting normal senescence.^2^ However, normal mice may develop teratomas or be lethal after continuous expression of all four factors, presumably because the originator of c-Myc reduces the mouse's lifetime by various cellular mechanisms. Consequently, the increasing evidence indicates that expressing only Oct4, Sox2, and Klf4 (OSK) is a safer method for *in vivo* reprogramming to delay aging instead of OSKM.^3^ Although the Yamanaka factor remains the predominant induction method, its induced pluripotent stem cells are susceptible to carcinogenesis, which makes it difficult for direct *in vivo* gene therapy. Therefore, optimizing the reprogramming system for anti-aging *in vivo* necessitates addressing key challenges, such as replacing oncogenic transcription factors. In this study, we generated a novel partial reprogramming method by the combination of OSK and TERT and reported that it could significantly postpone cellular senescence through the genomic alternation.

Human embryonic lung MRC-5 fibroblasts are a typical cell model that closely resembles human cells in their natural state, making them valuable for studying cell senescence and understanding the mechanisms of human cell aging. Normal fibroblasts can only proliferate a limited number of times (∼50 populations) *in vitro*, which is termed the Hayflick limitation. To evaluate the anti-aging effect of OSK and TERT gene therapy, in this study, we first generated the OSK- and TERT-expressing plasmids using a pcDNA3.3 vector. The constructed plasmid profile and confirmation results are shown in Figure S1. Subsequently, MRC-5 cells were cultured and passed through 50 generations (the onset of replicative cellular senescence) and then transfected with OSK-expressing plasmid with or without TERT-expressing plasmid. The relative expression levels of youth or aging-related genes were confirmed by quantitative reverse transcription PCR and western blotting when cells passed into 60 generations. The results showed that the mRNA levels of youth-related genes, including OCT4, SOX2, Klf4, Nanog, c-Myc, and TERT were markedly increased (Fig. 1A; *P* < 0.01), while the expression of aging-related genes, including p16, p21, ZSCAN4, ATF3, and MMP13, and the expression of the inflammatory cytokine IL-6 were significantly reduced (Fig. 1B; *P* < 0.05). Meanwhile, similar results were also confirmed when cells passed into 70 generations (Fig. S2). Moreover, we found that the protein levels of TERT, Oct4, Sox2, Klf4, Zscan4, Nanog, and c-Myc were consistent with their mRNA expression levels (Fig. 1C, D; *P* < 0.05).

**Figure 1:**
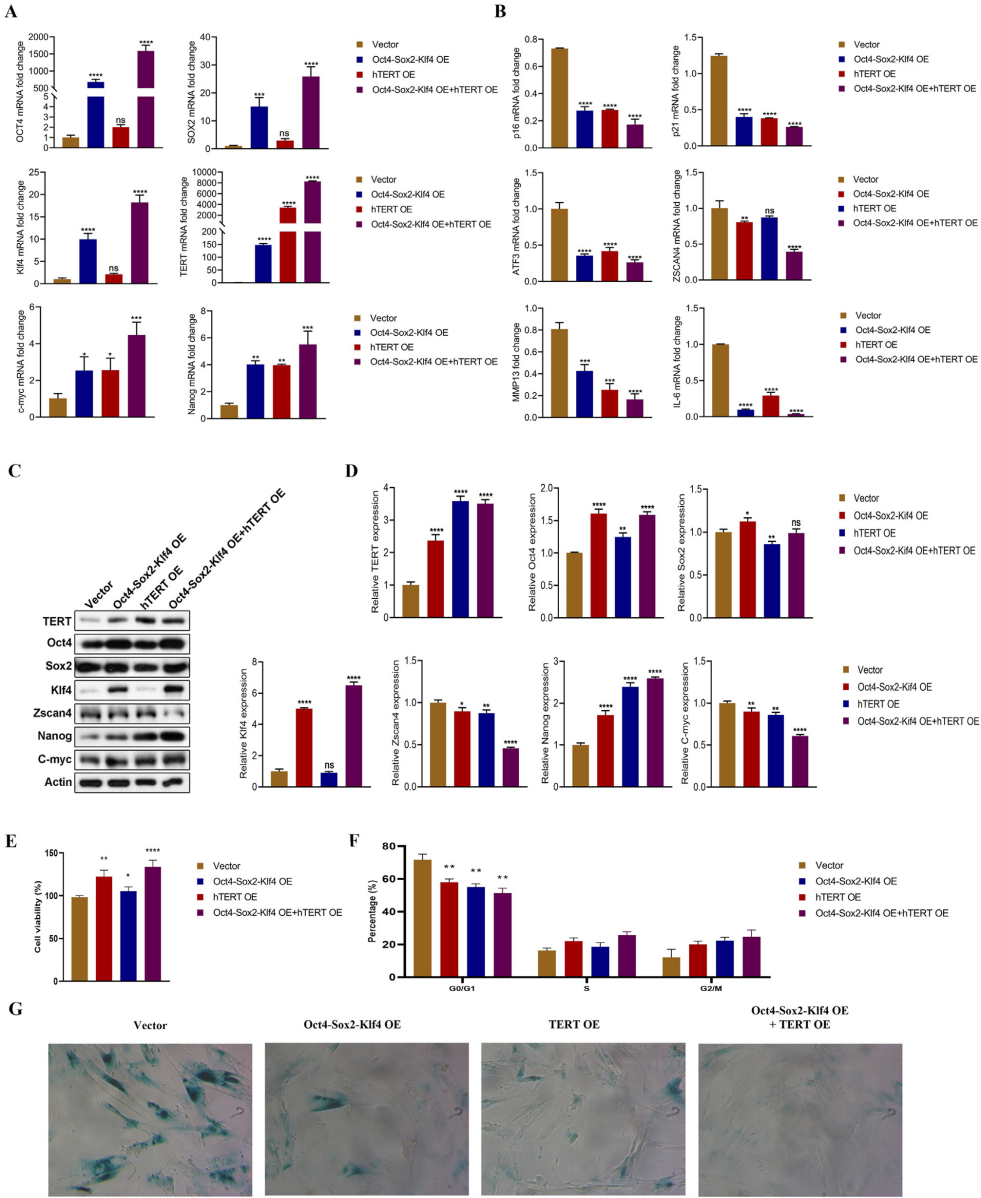
Expression of OSK and TERT promotes cell viability and reduces cellular senescence phenotype by modulating the aging-associated genes. The human lung fibroblast MRC-5 was transfected with OSK and/or TERT overexpressing plasmids when passed through 50 generations. Then, the cells were cultured into 60 generations, and quantitative reverse transcription PCR and western blotting were performed to analyze the expression of youth and aging-related genes. Moreover, the changes of cell viability and phenotype were also examined. **(A)** The mRNA levels of youth-related genes Oct4, Sox2, Klf4, Tert, c-Myc, and Nanog. **(B)** The expression of aging-related genes, including p16, p21, ZSCAN4, ATF3, MMP13, and the inflammatory cytokine IL-6. **(C)** Western blot image of the expression of TERT, Oct4, Sox2, Klf4, Zscan4, Nanog, and c-Myc. **(D)** The relative gray value of (C). **(E)** Summarized data indicated the cell viability performed by CCK-8 assay. **(F)** Summarized data indicated the cell cycle analysis performed by propidium iodide staining. **(G)** β-galactosidases staining assay indicated the expression of β-galactosidases. The summarized data shown were represented as mean ± standard deviation. The data were representative of three independent experiments (*n* = 3). The statistical significance of comparisons among multiple groups was analyzed by one-way ANOVA in comparison with the control vector (∗*P* < 0.05, ∗∗*P* < 0.01, and ∗∗∗*P* < 0.001).

In the next, we further detected the changes of cell phenotype after transfected with OSK- and TERT-expressing plasmids in MRC-5 cells at 50 generations. The cell counting kit-8 (CCK-8) assay was performed to examine the cell viability when cells passed into 60 generations. The results showed that combined overexpression of OSK and TERT significantly improved the cell viability, compared with single treatment (Fig. 1E; *P* < 0.05). Since cellular senescence stems from the persistent arrest of the cell cycle, we then examined the cell cycle by conducting propidium iodide staining. The results indicated that the G0/G1 phase was markedly decreased by combined overexpression of OSK and TERT in MRC-5 cells at 60 generations (Fig. 1F; *P* < 0.01), while the S phase and G2 phase increased to some extent with no significant difference. Moreover, β-galactosidase staining assay indicated that the expression of β-galactosidases was also markedly decreased after the co-expression of OSK and TERT at 60 generations of MRC-5 cells (Fig. 1G). As predicted, similar results were also confirmed when cells passed into 70 generations (Fig. S3). Therefore, all the results suggest that the overexpression of OSK and TERT in MRC-5 cell models may delay cellular senescence by modulating the expression of aging-related genes, thereby maintaining cell viability and delaying the aging phenotype significantly.

In this study, we have confirmed that the optimized OSKT (combined OSK and TERT therapy) method is potentially more effective than either OSK or TERT therapy alone *in vitro*. Previous research has demonstrated that the expression of OSK can restore youthful DNA patterns, promote axon regeneration, and improve vision in aged mice,^4^ while TERT gene therapy has been shown to significantly increase the longevity of 1- and 2-year-old mice *in vivo*.^5^ Collectively, this evidence supports the proof of concept for combining OSK and TERT to delay cellular aging. Although our findings currently validate this potential only in a replicative cell senescence model *in vitro*, they suggest the possibility of extending the lifespan of aging mice, which we aim to investigate in future studies. Furthermore, this gene reprogramming approach provides a robust theoretical foundation for its application in other cell types, such as aging immune cells, neurons, and other somatic cells, in anti-aging research.

Recent evidence increasingly suggests that aging is associated with a progressive decline in physiological functions and an elevated risk of various age-related diseases, including diabetes, neurodegenerative disorders, cardiovascular diseases, and cancer. Notably, gene therapy has demonstrated potential in effectively decelerating the aging process across multiple organs and systems, thereby reducing the incidence and severity of age-related diseases. Consequently, identifying potential strategies to delay cellular senescence is crucial for advancing the prevention and treatment of neurodegenerative diseases and cancer. Despite this, exploring novel optimal alternative methods to the OSKM guidance system is still the main research direction in the field of anti-aging and regenerative medicine.

In conclusion, our research study provides a novel potential composed method of OSK and TERT gene therapy. The optimized partial reprogramming system would be a safer and more efficient way to promote anti-aging research in clinical application and development.

## CRediT authorship contribution statement

**Mengmeng Jiang:** Writing – original draft, Formal analysis, Data curation. **Qianqian Xu:** Visualization, Validation, Methodology. **Zhengzhi Wu:** Writing – review & editing, Project administration, Funding acquisition, Conceptualization.

## Ethics declaration

This research does not contain clinical and animal experiments.

## Funding

This work was supported by the Major New Drug Innovation Project of the Ministry of Science and Technology of China (No. 2017ZX09301001), the 10.13039/501100001809National Natural Science Foundation of China (No. 81574038, 81473742), the China Central Finance Improvement Project for the National Key Laboratory of Chinese Medicine (China Central Finance, CS [2021] No. 151), the Shenzhen Science and Technology Program (China) (No. JCYJ20220818101806014), and the Team-based Medical Science Research Program (China) (No. 2024YZZ11).

## Conflict of interests

The authors declare that they have no known competing financial interests relevant to this review.
