# Supplementary material for: Optimized Yamanaka factors combined with TERT gene therapy for enhanced anti-aging effects
Source: Genes Dis. 2025 May 12;13(2):101669. doi: 10.1016/j.gendis.2025.101669 (PMC12617746; doi:10.1016/j.gendis.2025.101669)
Supplement: Multimedia component 1 [file mmc1.docx]

Supplementary Materials for

**Optimized Yamanaka Factor combined TERT gene therapy for enhanced anti-aging effects**

Mengmeng Jiang, Qianqian Xu, Zhengzhi Wu

Corresponding author:

Zhengzhi Wu, Foreign academician of the Ukrainian National Academy of the National Academy of Engineering Sciences, Shenzhen, China.

Email: szwzz001@163.com

**The PDF file includes:**

Materials and methods

Figure S1. OSK and TERT-expressing plasmids profile.

Figure S2. Overexpression of OSK and TERT changes the expression of aging-related genes in MRC-5 cells at 70 generations.

Figure S3. Overexpression of OSK and TERT changes the viability and phenotype of MRC-5 cells at 70 generations.

**Materials and methods**

**Cell culture**

Human embryonic lung MRC-5 fibroblasts were purchased from ATCC, and culture in DMEM complete medium, containing 10% FBS (Gibco) and 1% penicillin /streptomycin, at 37℃, 5% CO_2_ incubator. The cells were passed every three days for one generation, when reached at 50 generations, the cells were prepared to use.

**Plasmid construction and cell transfection**

The Oct4-Sox2-Klf4 and TERT overexpressing plasmids were separately constructed into pcDNA3.3 vector, then confirmed by agarose gel electrophoresis and second-generation sequencing. Lipofectamine 3000 was used to transfect the Oct4-Sox2-Klf4 and/or TERT overexpressing plasmids into MRC-5 cells, when cells were cultured and passage to 60 generations or 70 generations, the cells were harvested and prepared to analysis. All experimental protocols were conducted according to the manufacturer instructions.

**RT-qPCR analysis**

After transfection of Oct4-Sox2-Klf4 and/or TERT overexpressing plasmids. Total RNA was isolated from MRC-5 cells at 60 or 70 generations by Trizol reagent then transcribed into cDNA by PrimeScript RT reagent kit with gDNA Eraser (Takara, Cat# RR047A). Real-time PCR was performed by SYBR Green PCR kit according to the manufacturer specifications. β-actin was used as referenced gene to calculate the ΔCT value. The 2^-ΔΔCт^ method was used to evaluate gene expression. The primers used were shown as follows:

| **Gene name** | **Primer sequences (Forward 5'-3')** | **Primer sequences (Reverse 5'-3')** |
| --- | --- | --- |
| Oct4 | GAAGGATGTGGTCCGAGTGT | GTGACAGAGACAGGGGGAAA |
| Sox2 | AACCCCAAGATGCACAACTC | CGGGGCCGGTATTTATAATC |
| Klf4 | CCCACACAGGTGAGAAACCT | ATGTGTAAGGCGAGGTGGTC |
| Tert | GCGTTTGGTGGATGATTTCT | CAGGGCCTCGTCTTCTACAG |
| C-myc | AGAGAAGCTGGCCTCCTACC | CGTCGAGGAGAGCAGAGAAT |
| Nanog | ACGAGGTCAGGAAATCGAGA | TCTTGCAGCTCCTTTTCGTT |
| Lap2a | CGGAGTGAATCCTGGTCCTA | TGCTCTGCCCTTTAGTGGTT |
| p16 | CTTCCTGGACACGCTGGT | TTCTTTCAATCGGGGATGTC |
| p21 | GACACCACTGGAGGGTGACT | CAGGTCCACATGGTCTTCCT |
| Atf3 | GTGCCGAAACAAGAAGAAGG | TGGAGTCCTCCCATTCTGAG |
| Btg2 | AAGATGGACCCCATCATCAG | AGCACTTGGTTCTTGCAGGT |
| Zscan4 | CACAGGAATGAGAGGCCATT | TATGTGGATGACTGGCGGTA |
| Mmp13 | AACATCCAAAAACGCCAGAC | GGAAGTTCTGGCCAAAATGA |
| IL-6 | AGGAGACTTGCCTGGTGAAA | CAGGGGTGGTTATTGCATCT |

**Western blotting analysis**

The harvested MRC-5 cells were lysed in RIPA buffer containing proteinase inhibitor cocktails. The protein samples were separated through SDS-PAGE, then transferred to PVDF membrane. The non-specific proteins were blocked by 5% milk, then incubated with indicated primary antibody, and HRP-conjugated secondary antibody (Cell Signaling Technology, USA). The following antibodies were used: TERT (1:1000, #ab32020, Abcam), OCT4 (1:2000, #2750S, CST), SOX2 (1:2000, #23064, CST), KLF4 (1:500, #12173, CST), ZSCAN4 (1:1000, #ab153865, Abcam), NANOG (1:500, #4903S, CST), C-MYC (1:1000, #18583S, CST), β-Actin (1:2000, #3700, CST).

**Cell proliferation assay**

After transfection of Oct4-Sox2-Klf4 and/or TERT overexpressing plasmids, MRC-5 cells were cultured to 60 or 70 generations, then seeded into flat-bottomed 96-well plate at a density of 10000 cells/well in an appropriate culture medium. The cell viability was examined using Cell Counting Kit-8 (CCK-8) assay. 10μl of CCK-8 (5 mg/ml) was added into the 96 well plates and incubate for another 4h at 37℃ in the incubator. Finally, the cell viability was measured by the absorbance at 450 nm using a microplate reader.

**Cell cycle assay**

After transfection of Oct4-Sox2-Klf4 and/or TERT overexpressing plasmids, MRC-5 cells were cultured to 60 or 70 generations, then seeded into 6-well plate at a density of 5◊10^5^ cells/well in an appropriate culture medium. After 24h, the cells were harvested and washed one time with 1◊PBS, then fixed by 70% ethanol at -20℃ for 16 hours. Finally, the cells were washed and stained with PI (10 μg/ml) according to the standard protocol. Flow cytometry was performed to detect the cell cycle, calculate the percentage of G1/G0, S, and G2/M phase.

**Senescence-associated β-Galactosidase assay**

The Senescence-β-Galactosidase (SA-β-Gal) Staining Kit (Solarbio life sciences, Cat#G1580) was used to detect cell aging. According to the manufacturer’s instruction, 1×10^5^ MRC-5 cells were seeded in a 12-well plate, and fixed for 15min with fixation buffer at room temperature. After washed with PBS for three times, cells were incubated in SA-β-Gal–staining solution at 37℃ in a dry incubator (without CO_2_) overnight. The senescent cells were dyed blue green. The percentage of senescent cells was measured using the following formula: (positively stained cells/total cells)×100%.

**Statistical analysis**

All in vitro experiments were performed at least two or three times. Comparisons between two groups were analyzed by student’s t test, and the multiple groups comparison were analyzed by one-way ANOVA test using GraphPad Prism 8.3.0 (GraphPad, San Diego, CA, USA). When conducting these parametric tests, the Shapiro-Wilk test was performed to assess normality. For samples with heteroscedasticity, the Mann-Whitney U test and Kruskal-Wallis test were utilized to evaluate differences. The summarized data were represented as mean ± standard deviation (SD) from at least two independent experiments. *P* value ≤0.05 determines a statistically significant difference.

**
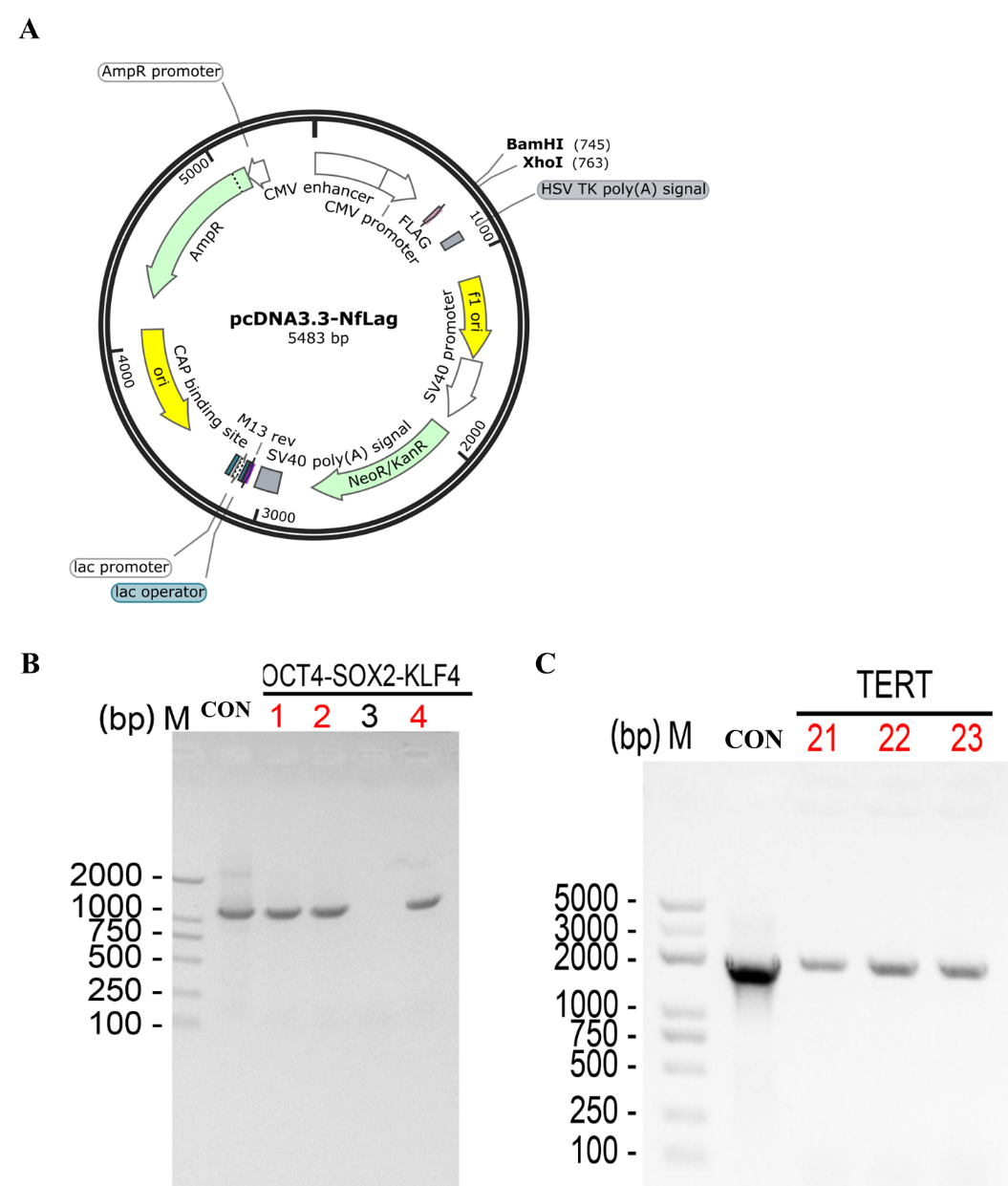
**

**Fig. S1. OSK and TERT-expressing plasmid profile.** To clarify the anti-aging effect of optimal Yamanaka factor with TERT gene therapy, we first utilized the pcDNA3.3 vector to construct the Oct4-Sox2-Klf4 and TERT overexpressing plasmids. A, the plasmid profile of pcDNA3.3. B, agarose gel electrophoresis showed the amplification of Oct4-Sox2-Klf4 in plasmid. C, agarose gel electrophoresis showed the amplification of TERT in plasmid.


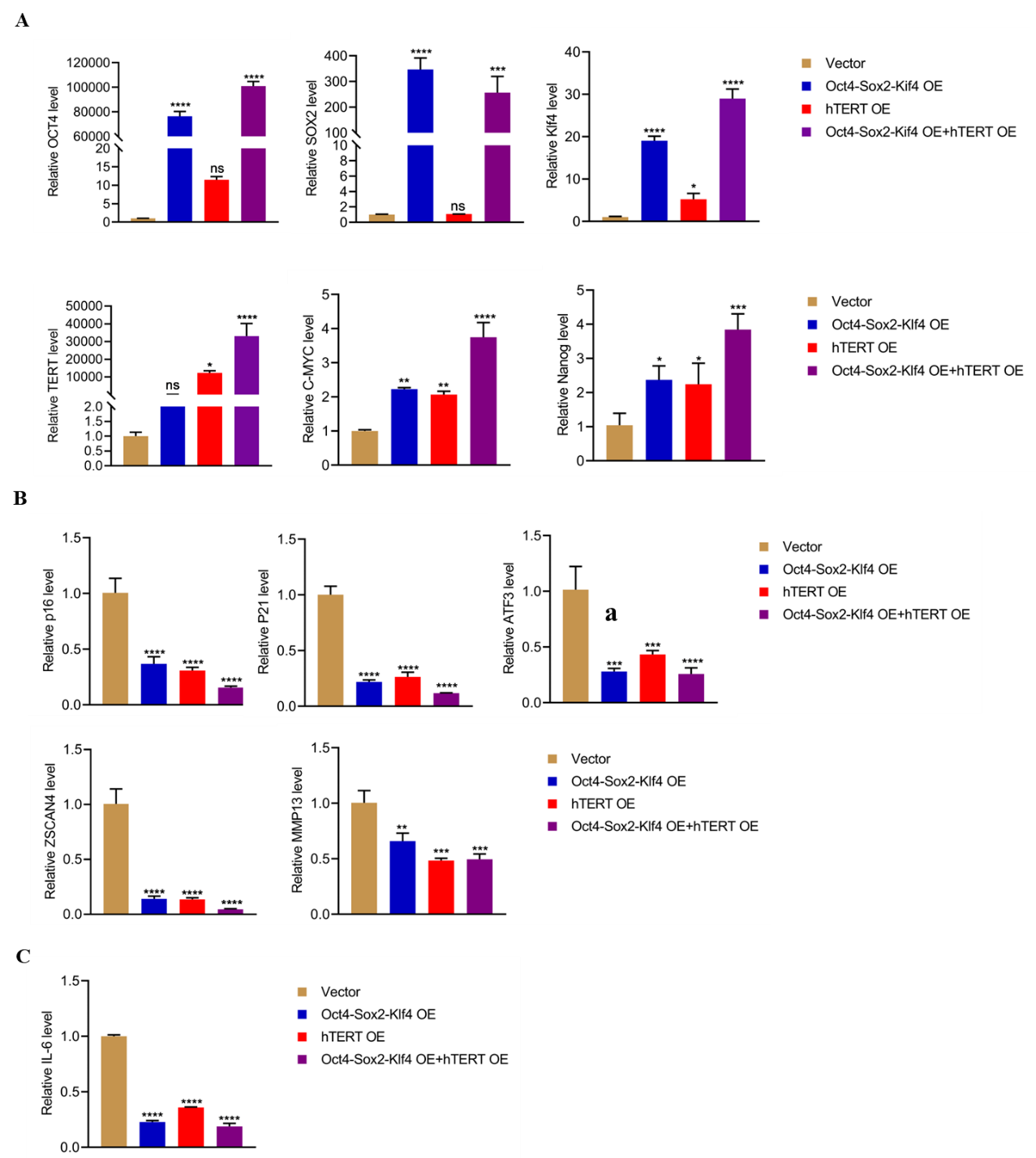


**Fig. S2.** **Overexpression of OSK and TERT changes the expression of aging-related genes in MRC-5 cells.** The human lung fibroblasts MRC-5 was transfected with OSK and TERT overexpressing plasmids when passage to 50 generations, respectively. Then, cultured the cells into 70 generations, RT-qPCR was performed to analyze the expression of youth and aging-related genes. A, the mRNA levels of youth-related genes Oct4, Sox2, Klf4, Tert, Nanog, and C-myc. B, the expression of aging-related genes, including p16, p21, ZSCAN4, ATF3, and MMP13. C, the expression of inflammatory cytokine of IL-6. Summarized data (mean ± SD) shown were representatives of three independent experiments with similar results. The statistical significance among multiple groups comparison was analyzed by one-way ANOVA, by comparison with the control vector, * P<0.05, ** P<0.01, *** P<0.001.


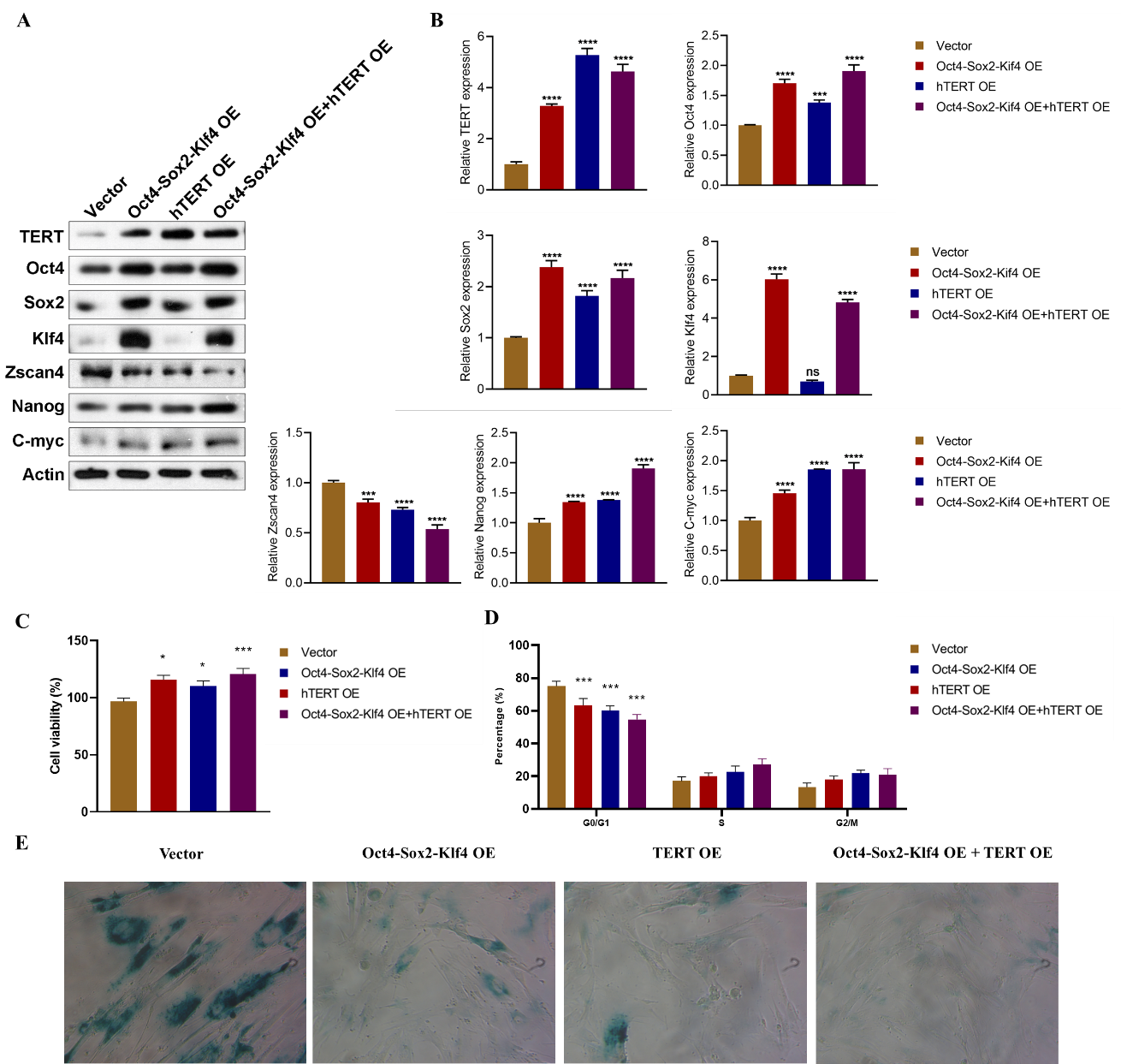


**Fig. S3.** **Overexpression of OSK and TERT changes the viability and phenotype of MRC-5 cells.** After transfection of Oct4-Sox2-Klf4 and/or TERT overexpressing plasmids, MRC-5 cells were cultured to 70 generations. The protein levels of some relative genes were confirmed by western blot, and the changes of cell viability and phenotype were also examined. A, western blot image of expression of TERT, Oct4, Sox2, Klf4, Zscan4, Nanog, and C-myc. B, the relative gray value of A. C, CCK-8 assay was used to examine the cell viability. D, cell cycle analysis. E, β-galactosidases staining assay indicated that the expression of β-galactosidases. The summarized data shown were represented as mean ± SD. The results data representative of three independent experiments (n=3). The statistical significance among multiple groups comparison was analyzed by one-way ANOVA, by comparison with the control vector, * P<0.05, ** P<0.01, *** P<0.001.
